# The frequency of periodontitis in end-stage renal disease on hemodialysis in a sample of Egyptian population: multi-center clinical cross-sectional study

**DOI:** 10.1186/s12903-021-02032-x

**Published:** 2022-01-03

**Authors:** Asmaa Abou-Bakr, Radwa R. Hussein, Eman Khalil, Enji Ahmed

**Affiliations:** 1 Oral Medicine and Periodontology, Faculty of Dentistry, Cairo University, Giza, Egypt; 2 Oral Medicine and Periodontology, Faculty of Dentistry, The British University in Egypt, El Sherouk City, Egypt; 3 Oral Medicine and Periodontology, Faculty of Dentistry, Ain Shams University, Cairo, Egypt

**Keywords:** Chronic renal disease, Hemodialysis, Periodontitis, Frequency

## Abstract

**Background:**

There is a general assumption that periodontal disease is highly prevalent among patients with chronic renal failure undergoing hemodialysis. The aim of the study to estimate the frequency of periodontitis in patients on hemodialysis among a sample of the Egyptian population, as well as the correlation between different clinical parameters of periodontal status with serum creatinine and blood urea. This may rule out the bidirectional relationship between periodontitis and renal failure in patients on hemodialysis.

**Methods:**

The study was conducted on 263 hemodialysis patients (165 males and 98 females) at three dialysis centers in Benha Governorate, Egypt (Benha Hospital, Tukh hospital, Qalyub hospital). Periodontal parameters including plaque index (PI), gingival index (GI), clinical attachment level (CAL), and probing pocket depth (PPD) had been recorded in these patients. Serum urea and creatinine levels had been measured, the data had been collected and undergone statistical analysis.

**Results:**

Frequency of periodontitis was 85.6% with stage III is the most prevalent stage. There was a significant positive strong correlation between age and periodontitis stage (r_s_ = 0.707, p < 0.001). There was a positive correlation between clinical parameters and serum creatinine level.

**Conclusion:**

In the present study, a high frequency of periodontitis had been found among ESRD patients on hemodialysis in the severe form (stage III) periodontitis. There was a significant direct correlation between the severity of periodontitis and CAL with a duration of hemodialysis. There was a weak insignificant association between periodontal indices (PD, BOP, and plaque score) and duration of hemodialysis.

## Introduction

Chronic kidney disease (CKD) is a progressive loss of kidney function and structural damage caused by a variety of factors. The glomerular filtration rate (GFR) (total amount of fluid filtered through all functioning nephrons per unit of time), has been used to determine the overall kidney function and diagnosis of CKD [1].

According to current international recommendations, the patient considered having CKD if a GFR of less than 60 ml/min per 1.73 m^2^ with signs of renal damage that last for at least three months [2]. CKD is the fourteenth largest cause of death, accounting for 12.2 fatalities per 100,000 people in 2012. The World Health Organization estimated that 864,226 fatalities had been affected by this ailment in 2012 [3].

People whose GFR falls below 15 ml/min per 1.73 m^2^ develop end-stage renal disease (ESRD). Renal function has deteriorated to the point where it can no longer sustain life over the long term, necessitating the introduction of renal replacement therapy (RRT) either dialysis or renal transplant but if the GFR is around 5–10% with a high level of uremia, then this condition requires renal dialysis [4].

Between the ages of 20 and 40, the first and most substantial peaks of ESRD occur and are frequently the result of CKD of unknown etiology (73%). The second peak occurs between the ages of 50 and 70, and CKD is frequently caused by diabetes and systemic arterial hypertension (59.6%). The patients' ages ranged from 20 to 30 years old (n 14 112), with focal segmental glomerulosclerosis (FSGS) being the most common diagnosis (54%) [5].

In Egypt, the statistics that had been calculated in (2014), found that the prevalence rate of ESRD was 366 pmp in the governorate of Assiut in upper Egypt [6]. The prevalence of ESRD had been reported to be 330 pmp in a cross-sectional study conducted on both sexes in Menoufia, another Egyptian governorate with a population of 2.2 million [7]. In the Sharkia governorate of Egypt, the cumulative prevalence of ESRD patients on maintenance hemodialysis was 442 per million people in 2017. (0.0442%). Between the ages of 50 and 59, the largest proportion of patients with ESRD was (30.8%). The majority of ESRD patients were from rural areas (60.4%) [8].

ESRD and the medications used by those patients create complications in a variety of systems and organs, which frequently worsens or causes new pathologies in the oral cavity, such as caries, periodontal disease, mucosal lesions, and decreased saliva output [9, 10].

The emergence of a chronic systemic inflammatory disease in people with ESRD is a common occurrence. The reasons of this inflammation are most likely multifaceted and complex. A number of illnesses and comorbidities have been identified as potential influencers of an increase in the inflammatory state [11, 12].

The accelerated periodontal disease with pocket formation, gingival recession, and bone and tooth loss is due not only to inadequate oral hygiene and inflammatory disease burden but also to renal osteodystrophy, high urea concentration, salivary changes in composition and the host factors related to the underlying systemic disease that modify the host response to periodontal infection [13, 14].

Periodontal disease is a chronic inflammatory condition that affects the tooth-supporting structures, resulting in clinical attachment loss due to an imbalance between the host response and the dental biofilm (bacterial plaque) [15].

Periodontal disease is caused by immunological and inflammatory mechanisms that induce a dysregulation in the host response due to periodontal bacteria superinfection [16]. In addition, CP has been linked to elevate levels of inflammatory biomarkers such interleukin 6, prostaglandin, and C-reactive protein (CRP) [17] in the blood. Endogenous inhibitor of nitric oxide (NO) metabolism and subsequent endothelium-dependent vasodilation has been discovered in asymmetric dimethylarginine (ADMA) [18]. ADMA has been demonstrated to operate as a competitive inhibitor of NO synthase, and elevated serum ADMA levels have been linked to a variety of metabolic diseases, including periodontitis [19].

According to Curro et al. [20], both TG2 and plasma FXIIIA preserve the expression levels seen in normal tissue among the TGs found in periodontal tissues. TG1 and TG3 isoforms are significantly downregulated in periodontal disease.

As a result, Smith et al. [21], concluded that alterations in TG expression could be linked to increased MMP expression, which is likely due to active inflammation in gingival epithelial cells throughout periodontal disease.

Severe periodontal disease was the 11th most common disease worldwide, according to the Global Burden of Disease Study (2016) [22]. Periodontal disease has been found to affect anywhere from 20 to 50% of people worldwide [23]. It’s one of the most common causes of tooth loss, and it can affect mastication, appearance, self-esteem, and quality of life [24].

In 2016, periodontal diseases were responsible for 3.5 million years lived with disability (YLD) globally [22]. The global prevalence of periodontal disease is predicted to rise in the coming years as the world's population ages and patients desire to keep their natural teeth [24]. Hemodialysis patients have been found to have severe periodontal disease [25]. Periodontitis itself causes systemic inflammation and has been linked to poor hemodialysis outcomes [26].

To our knowledge, there were no published reports about the prevalence of periodontitis in patients with ESRD among the Egyptian population according to the new classification of periodontal and peri-implant diseases (2017). Consequently, the purpose of this study was to determine the prevalence and severity of periodontitis in Egyptian patients with ESRS who were undergoing hemodialysis, as well as the correlation between different clinical parameters of periodontal status with serum creatinine and blood urea. This may rule out the bidirectional relationship between periodontitis and renal failure in patients on hemodialysis.

## Subjects and methods

### Study design

This multicenter-clinical-cross-sectional study had been conducted on 263 patients at three dialysis centers in Benha Governorate, Egypt (Benha Hospital, Tukh hospital, Qalyub hospital) by using direct interview, dental and periodontal examination of ESRD patients, in addition to using medical records for medical data collections.

The proposal had been reviewed and approved by the Faculty of Dentistry, Ain Shams University Research Ethics Committee (FDASU-REC) with the final registration number: FDASU-REC M091810. Individual patient’s data had been kept confidential. The procedure was fully explained to the patients and all the recruited patients signed informed consent.

### Sample size calculation

Based on the prevalence of end-stage renal disease in Egypt to be 366 per million according to El Arbagy et al. [6] and considering the population of Egypt to be 90,000,000. According to Abbas et al. [27], the prevalence of stage II, III, and IV periodontal disease amongst Egyptians had been estimated as 15.2%, 4.4%, and 2.05% respectively, giving a total of 21,65%. Total sample size had been calculated to be 263 ESRD patients with a significance level (α error) set at 5%, power (1 − β error) at 90% for two-sided hypothesis test [28].

### Patient selection

#### Patients recruitment

The target sample had been reached through consecutive sampling of ESRD patients who visited the hemodialysis centers over a period of 3 months from March till May 2021 of the available numbers.

*Inclusion criteria* included both genders with an age range from 20 to 70 years according to El Arbagy et al. [6]; cases clinically diagnosed as having end-stage renal disease stages; all patients had been on hemodialysis for a minimum of 6 months and maximum of 2 years; frequency of dialysis was twice a week and duration of 3 h per session.

*Exclusion criteria* included patients with acute renal failure; patients with CKD under conservative treatment; patients with a history of any serious illness as malignancy or who undergo kidney transplant; patients who had periodontal therapy in the previous six months; smokers [29], as known that smoking is an important risk factor for periodontal disease [30], so we exclude them to minimize the confounders in our studied sample of ESRD; patients who refused to participate in the study.

Patients’ data included patient’s age, sex, duration of hemodialysis and any systemic disease other than end-stage renal disease. Medical records included updated serum creatinine and blood urea level.

#### Clinical periodontal examination

Two calibrated examiners (AA and EA) performed a full mouth periodontal examination and periodontal charts in addition to radiological examination for all the participants to determine the periodontal status and periodontitis stage according to the new classification of periodontal and peri-implant diseases (2017) as: Periodontally healthy individuals are those who don’t have a loss of clinical attachment (due to periodontal disease), pocket depth less than or equal to three mm, and the scores of bleeding on probing in the whole mouth less than 10%; gingivitis patients are those with no loss of clinical attachment (due to periodontal disease), pocket depth less than or equal to three mm, and the scores of bleeding on probing in the whole mouth is more than 10% [31].

Patients with periodontitis had at least two non-adjacent teeth. Stage I periodontitis had 1–2 mm interdental CAL with radiographic bone loss affecting less than 15% of root length and no teeth lost due to periodontitis. Stage II periodontitis had 3–4 mm interdental CAL with radiographic bone loss affecting 15–33% of root length and no teeth lost due to periodontitis. Stage III and IV periodontitis had ≥ 5 mm interdental CAL with radiographic bone extending to middle or apical third of the root. In stage III periodontitis, a number of teeth lost due to periodontitis ≤ 4 whereas in stage IV they are > 5 teeth [32].

Plaque index (PI) [33] and gingival index (GI) [34] had been estimated to assess the periodontal and oral hygiene status of each participant in the study. The PI had been calculated by dividing the number of plaque-containing surfaces by the total number of available surfaces. The surfaces that did not show soft plaque accumulations at the dentogingival junction had not been included in the study.

With a periodontal probe, PD and CAL were measured on six locations of the teeth (mesio-buccal/facial, mid-buccal/facial, disto-buccal/facial, mesio-lingual/palatinal, mid-lingual/palatinal, disto-lingual/palatinal) excluding third molar.

Loss of clinical attachment generally is measured as the distance from the CEJ to the depth of pocket. When probing depth (distance from free gingival margin to the base of sulcus/pocket) was equal to the gingival margin (distance from free gingival margin to the CEJ) this means that there is no loss of clinical attachment and attachment epithelium is still attached to the CEJ. In other words, if during measuring the pocket depth the probe didn’t pass beyond the CEJ and stopped above this point, this means no loss of clinical attachment.

Tooth loss due to periodontitis was addressed through history taking from the patient. Patients provided history of sound teeth with great mobility that caused discomfort and difficulty of eating and mastication.

The proportion of bleeding sites 10 s after being stimulated by a standardized manual probe with a controlled force to the bottom of the sulcus/pocket at six locations (mesio-buccal, buccal, disto-buccal, mesio-lingual, lingual, disto-lingual) on all present teeth was assessed dichotomously as a BOP score on all present teeth.

Case definition of gingivitis according to Trombelli et al. [31] is:

- Probing depth ≤3 mm
- BOP score ≥10%, ≤30% if localized and >30% if generalized
- Absence of clinical attachment loss
- Absence of radiographic bone loss

### Statistical analysis

Numerical data were presented as mean and standard deviation values. Categorical data were presented as frequencies (n) and percentages (%) and were analyzed using Fisher’s exact test. Numerical correlations were analyzed using Spearman’s correlation coefficient. The significance level was set at p ≤ 0.05 for all tests. Statistical analysis was performed with R statistical analysis software version 4.1.1 for Windows.^1^

### Regression models

Two separate linear regression models were built to evaluate the effect of each of the studied periodontal parameters (as the independent variable) on blood urea level, serum creatinine, sex, age and duration of hemodialysis (as the dependent variable). The deviance goodness-of-fit test indicated that the model was a good fit to the observed data, χ^2^(7) = 164.80, p = 1 and the final model statistically significantly predicted the dependent variable over and above the intercept-only model, χ^2^(7) = 317.01, p < 0.001.

## Results

### Demographic data

This cross-sectional study was conducted on 263 patients with end-stage renal disease on hemodialysis with a mean age of (48.12 ± 9.80) years. 62.7% (165) the patients were males and 37.3% (98) were females with p value < 0.001.

### Frequency of periodontitis in ESRD

From the total sample of patients comprising 263 patients, the prevalence of periodontitis was 85.6% (225) patients with 14.4% (38) only had gingivitis.

**Fig. 1:**
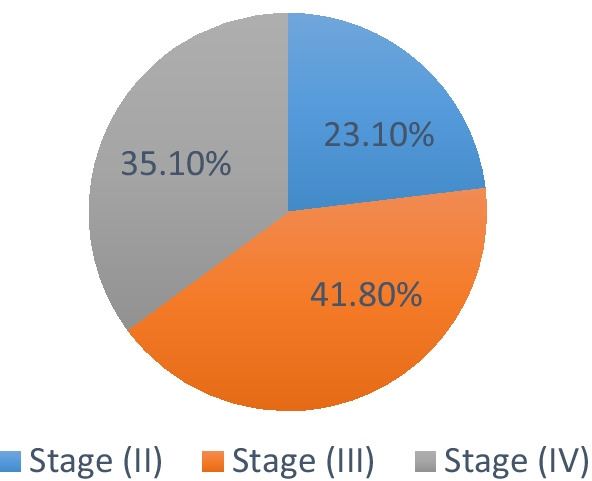
Pie chart showing prevalence of different stages of periodontitis in ESRD

Frequency and percentage of periodontitis stages in ESRD were shown in Fig. 1. Stage (III) was the most prevalent stage of periodontitis with 41.8% (94) followed by a stage (IV) 35.1% (79) with no significant difference between them, while the least prevalent stage was stage (II) 23.1% (52) being significantly lower than stages III and IV as in Fig. 1.

### Periodontitis and medical conditions

The most prevalent medical condition in periodontitis patients with ESRD undergoing hemodialysis was hypertension with 50.1% (191) patients, followed by 21.5% (82) diabetic patients, 16.5% (63) patients with virus C infection, 5.5% (21) patients with secondary hyperparathyroidism, 3.9% (15) patients with idiopathic renal disease and 2.4% (9) patients with cardiac conditions.

The prevalence of medical conditions in each stage of periodontitis was presented in Table 1. In hypertensive patients, 23.4% of patients had stage II periodontitis, 39.8% had stage III periodontitis and 36.8% of patients had stage IV periodontitis. In patients with secondary hyperparathyroidism, 40.0% of patients had stage II periodontitis, 6.7% had stage III periodontitis and 53.3% of patients had stage IV periodontitis. In patients with Virus C infection, 21.3% of patients had stage II periodontitis, 34.4% had stage III periodontitis and 44.3% of patients had stage IV periodontitis. In diabetic patients, 10.7% of patients had stage II periodontitis, 57.3% had stage III periodontitis and 32% of patients had stage IV periodontitis.

**Table 1:** Descriptive data of ESRD patients with different stages of periodontitis

Parameter	Total sample			Periodontitis stage			p value
	Mean ± SD			Stage (II)	Stage (III)	Stage (IV)	
Age	48.12 ± (9.80)			0.707			< 0.001*
Sex		Male	N%	30	57	53	0.509
				21.4%	40.7%	37.9%	
		Female	N%	22	37	26	
				25.9%	43.5%	30.6%	
*Medical condition*							
Hypertension			N%	40	68	63	
				23.4%	39.8%	36.8%	
Secondary hyperparathyroidism			N%	6	1	8	
				40.0%	6.7%	53.3%	
Virus C			N%	13	21	27	
				21.3%	34.4%	44.3%	
Diabetes mellitus			N%	8	43	24	
				10.7%	57.3%	32.0%	
*Clinical parameters*							
Mean probing depth (mm)	4.50 ± 0.74						
Mean CAL (mm)	6.30 ± 2.06						
Plaque %	24.74 ± 9.66						
BOP%	48.66 ± 13.65						
Number of teeth	3.51 ± 3.03						
% of sites with CAL ≥ 4 mm	33.79 ± 22.26						
% of sites with PD ≥ 3 mm	28.15 ± 20.00						
*Biochemical analysis*							
Duration of hemodialysis	16.01 ± 6.41						0.008*
Blood urea (mg/dl)	134.15 ± 33.44						
Serum creatinine (mg/dl)	4.54 ± 1.25						

### Correlation between different variables

The descriptive data of ESRD patients with different stages of periodontitis were presented in Table 1. There was no significant association between sex and stages of periodontitis (p = 0.509) (Table 1).

There was a significant positive strong correlation between age and periodontitis stage (r_s_ = 0.707, p < 0.001). The most advanced stage of periodontitis, which is stage IV, was more prevalent among patients of older ages and vice versa (Table 1).

There was a significant positive weak correlation between the age of the patients and the duration of hemodialysis (r_s_ = 0.177, p = 0.008). Older age patients have undergone hemodialysis for durations longer than young age patients. In the conclusion, there was a significant positive weak correlation between duration of hemodialysis and stage of periodontitis (r_s_ = 0.156, p = 0.013). The increase duration of hemodialysis is directly correlated with an increase in the severity of periodontitis (Table 1).

### Correlation between different periodontal clinical parameters and Blood Urea and serum creatinine level

Table 2 presents Mean ± SD values for clinical parameters (PD, plaque score, BOP, and deepest CAL), blood urea, and serum creatinine level.

**Table 2:** Correlation between different periodontal clinical parameters and blood urea and serum creatinine level

Parameter	Correlation coefficient (r_s_)	p value
*Mean probing depth (mm)*		
Blood urea (mg/dl)	0.089	0.182
Serum creatinine (mg/dl)	0.217	0.001*
*Plaque %*		
Blood urea (mg/dl)	0.178	0.001*
Serum creatinine (mg/dl)	0.018	0.786 ns
*BOP %*		
Blood urea (mg/dl)	0.324	< 0.001*
Serum creatinine (mg/dl)	0.045	0.504
*Mean CAL (mm)*		
Blood urea (mg/dl)	0.290	< 0.001*
Serum creatinine (mg/dl)	0.450	< 0.001*

^*^Significant (p ≤ 0.05); ns; non-significant (p > 0.05)

Correlation between different periodontal parameters (PD, plaque score, BOP and deepest CAL) and blood urea as well as serum creatinine level was presented in Table 2.

There was a significant positive correlation between mean probing depth and serum creatinine level (r_s_ = 0.217, p = 0.001) and a non-significant positive correlation between mean probing depth and blood urea level (r_s_ = 0.089, p = 0.182).

There was a significant positive correlation between plaque score and blood urea level (r_s_ = 0.178, p = 0.001) and non-significant positive correlation between plaque score and serum creatinine level (r_s_ = 0.018, p = 0.786).

There was a significant positive correlation between bleeding and probing score and blood urea level (r_s_ = 0.324, p ≤ 0.001) and a non-significant positive correlation between bleeding and probing score and serum creatinine level (r_s_ = 0.045, p = 0.504).

There was a significant positive correlation between deepest CAL and blood urea level (r_s_ = 0.290, p ≤ 0.001), as well as serum creatinine level (r_s_ = 0.450, p ≤ 0.001).

### Regression models analysis

An increase in age and in blood urea was associated with significantly increasing the odds of periodontal severity being higher with an odds ratio of 1.25(1.17–1.34) (p < 0.001) and 1.03(1.01–1.04) (p < 0.001) respectively. While an increase in serum creatinine level was associated with significantly lowering the odds of periodontal severity being higher with an odds ratio of 0.23(0.15–0.36) (p < 0.001).

Models showed age to have a significant positive effect on blood urea level (p < 0.001) and males to have a significantly higher blood urea levels than females (p = 0.013). All periodontal variables—except for probing depth- had a significant positive effect on blood urea level (p < 0.05). Results of regression models were presented in Table 3.

**Table 3:** Linear regression model between blood urea, sex, age and duration of hemodialysis (as dependent variable) and different periodontal parameters (as independent variable)

Outcome	Regression coefficient	Beta (95% CI) for regression coefficient		p value
		Lower	Upper	
Age	0.70	0.21	1.19	< 0.001*
Sex	− 11.35	− 20.30	− 2.93	0.013*
Mean probing depth (mm)	2.18	− 3.78	8.14	0.471 ns
Plaque %	0.59	0.14	1.04	0.010*
BOP %	0.73	0.43	1.04	< 0.001*
Mean CAL (mm)	4.36	2.30	6.42	< 0.001*
Number of teeth	3.34	1.95	4.73	< 0.001*
% of sites with CAL ≥ 4 mm	0.45	0.26	0.64	< 0.001*
% of sites with PD ≥ 3 mm	0.49	0.28	0.70	< 0.001*
Duration of hemodialysis	0.06	− 0.63	0.75	0.862 ns

*CI* confidence interval

Models showed age to have a significant negative effect on serum creatinine level (p < 0.001). All of periodontal variables—except for plaque percent and BOP percent—had a significant negative effect on serum creatinine level (p < 0.05). Results of regression models were presented in Table 4.

**Table 4:** Linear regression model between serum creatinine, sex, age and duration of hemodialysis (as dependent variable) and different periodontal parameters (as independent variable)

Outcome	Regression coefficient	Beta (95% CI) for regression coefficient		p-value
		Lower	Upper	
Age	− 0.06	− 0.07	− 0.04	< 0.001*
Sex	0.09	− 0.25	0.43	0.608 ns
Mean probing depth (mm)	− 0.39	− 0.61	− 0.17	< 0.001*
Plaque %	− 0.002	− 0.02	0.02	0.803 ns
BOP %	− 0.006	− 0.02	0.01	0.356 ns
Mean CAL (mm)	− 0.24	− 0.31	− 0.16	< 0.001*
Number of teeth	− 0.22	− 0.26	− 0.17	< 0.001*
% of sites with CAL ≥ 4 mm	− 0.03	− 0.03	− 0.02	< 0.001*
% of sites with PD ≥ 3 mm	− 0.03	− 0.03	− 0.02	< 0.001*
Duration of hemodialysis	− 0.01	− 0.03	0.02	0.532 ns

*CI* confidence interval

Table 5 reported the descriptive data in ESRD patients with gingivitis.

**Table 5:** Descriptive data of ESRD patients with gingivitis

Parameter	Value	N	N (%) Mean ± SD
Sex (n = 38)	Male	25	65.8%
	Female	13	34.2%
Age		–	35.61 ± 4.39
Medical condition (n = 44)	Secondary Hyperparathyroidism	6	13.6%
	Diabetes	7	15.9%
	Hypertension	20	45.5%
	Idiopathic renal disease	9	20.5%
	Virus C	2	4.5%
Duration of hemodialysis		38	8.26 ± 2.10
Blood urea (mg/dl)		38	97.63 ± 12.94
Serum creatinine (mg/dl)		38	4.04 ± 1.22
Mean probing depth (mm)		38	1.84 ± 0.75
Plaque %		38	13.68 ± 5.40
BOP %		38	25.50 ± 8.91
Mean CAL (mm)		0	0.00 ± 0.00
Number of missing teeth		0	0.00 ± 0.00
% of sites with CAL ≥ 4 mm		0	0.00 ± 0.00
% of sites with PD ≥ 3 mm		8	13.26 ± 5.14

Data are presented as N (%) or mean ± standard deviation

## Discussion

Periodontal disease is a chronic inflammatory disease caused by interaction between pathogenic anaerobic bacteria in dental plaque biofilm and host immune-inflammatory reaction. Periodontal disease is characterized by destruction in the tooth-supporting structures and alveolar bone resorption [35].

Several studies have described the association between periodontal disease and chronic kidney disease especially those on hemodialysis. Nowadays, there is a general agreement on the high prevalence of periodontal disease among patients undergoing hemodialysis compared to their healthy counterparts [36].

Owing to debilitation, poor immune response, and persistent inflammation, maintenance hemodialysis (HD) patients are more prone to infections. Owing to the illness and infectious diseases, the inflammation, which is also present in the mouth, can become more severe [37].

Our results demonstrated that there was a higher prevalence of males (62.7%) than females (37.3%) among the participants. This fact was compatible with earlier studies, which reported the higher prevalence among males in CKD patients with prevalence 61%, 64%, receptively [38, 39]. Male patients show a significantly higher prevalence of CKD and frequency rate of ESRD than those observed in female patients [40, 41]. Furthermore, diabetic males have a higher risk of renal failure than do diabetic females [42].

In the present study, the mean age of (48.12 ± 9.80) years which was in agreement with previous studies with mean ages of (47.9 ± 15.3) and (47.7 ± 4.9) years [43].

The frequency of periodontitis among hemodialysis patients was 85.6% (225 out of 263) patients. This was in accordance to Kim et al. [44] who reported that more than 99% of the renal patients on hemodialysis who had been evaluated in their study (115 patients) had periodontitis (107 patients). Similarly, Cholewa et al. [45] found a 100% prevalence of periodontitis among 103 HD patients with only one had a healthy periodontium. Many other previous studies among the patients with renal failure on hemodialysis reported similar results [46, 47].

Stage III periodontitis (severe periodontitis) was the most prevalent form of periodontitis among our sample with 41.8% (94 out of 263). This fact was in accordance with another study that reported high severity of periodontal disease in CKD patients on hemodialysis was associated with higher bacterial load compared with systemically healthy counterpart [48].

It has been suggested that increased severity and progression of periodontal disease is due to the compromised immunity in patients on hemodialysis specially the diabetic ones [49]. With poor oral hygiene status and nonstop plaque accumulation, this week immune system becomes unable to counteract the virulent plaque bacteria. In addition to other factors that can contribute to periodontal disease severity including high salivary urea concentration, and low salivary pH levels [50].

Furthermore, neglecting oral hygiene secondary to gingival bleeding is probably due to persistent inflammation of periodontal tissues in HD patients [51]. In conclusion, the chronic inflammation and constant bacterial challenge combined with compromised immune responses was accused of an extension of the inflammation from periodontal tissues into the bloodstream, and to the subsequent systemic inflammation in patients with HD [36].

In the current study, there was a significant positive strong correlation between age and periodontitis stage (r_s_ = 0.707, p < 0.001). The most advanced stage of periodontitis, which is stage IV, was more prevalent among patients with older ages and vice versa and this fact was in agreement with another study [43]. The alterations in the periodontal structure related to the patient age support that age may be a possible risk indicator for periodontal disease progression [52].

Our present study showed that hypertension and diabetes mellitus were the most frequent medical problems (50.1% and 21.5% respectively). Correspondingly, Segelnick and Weinberg [53] reported that hypertension and diabetes mellitus were the first two common diseases in their study on chronic renal patients.

Our results showed the frequency of periodontitis with various degrees of severity among hypertensive patients on hemodialysis. Previous studies had documented that hypertensive patients showed more severe periodontal conditions than do healthy one [54]. Also, it is well known that hypertension and periodontitis share common risk factors that may explain the link including tobacco smoking, stress, aging, and socioeconomic factors [55]. Severe periodontitis was found to be associated with major endothelial dysfunction that is reversible after successful periodontal therapy in hypertensive patients [56].

Our study should 57.3% of diabetic patients had stage III periodontitis (severe periodontitis). This was in accordance with Schmalz et al. [57], who reported a high prevalence of severe form periodontitis in diabetic patients on hemodialysis. Nowadays, there is an overall assumption that DM is a major cause of dialysis in patients with renal failure. That’s to say, many patients on hemodialysis have been receiving treatment for diabetes several years before. At the same time, diabetes has been considered an important risk factor for severe forms of periodontal disease [58]. Therefore, it’s logical to presume that hemodialysis patients with diabetes have more risk of developing periodontal disease than their non-diabetic matching group [36].

Diabetes mellitus type 2 and periodontitis are considered chronic diseases related to routines and lifestyle habits as well as socioeconomic factors and both have been correlated with systemic general inflammation [59]. It is presumed that there is a bi-directional association between periodontitis and diabetes mellitus type 2 [59].

As most of the ESRD patients on hemodialysis have complicated medical conditions such as having hypertension, diabetes, and increased hemorrhagic tendencies, these could play-act as important confounders in giving a final conclusion in the diagnosis of periodontal disease severity. It can also be reasonable that poor oral hygiene and its cumulative effect over the years might have caused increased severity periodontal tissue breakdown in these patients. Regular monitor of periodontal conditions over a successive period of time, right from the start of hemodialysis could give more expressive and meaningful results [47].

5.5% of periodontitis patients in the current study have secondary hyperparathyroidism with 53.3% had advanced periodontitis. Secondary hyperparathyroidism and vitamin D3 deficiency are observed in HD patients as possible common complications. Furthermore, they can lead to disruption in calcium hemostasis and linked decrease in bone mineral density and alveolar bone resorption [36].

In the present study, we found a significant positive weak correlation between the duration of hemodialysis and stage of periodontitis and deepest CAL (r_s_ = 0.156, p = 0.013). The increased duration of hemodialysis was directly correlated with an increase in the severity of periodontitis. This was in agreement with previous studies that showed a direct positive association between longer dialysis duration and periodontal clinical parameters in patients on hemodialysis [60, 61].

While our finding was not in line with previous studies that found no significant association between hemodialysis duration and the prevalence and severity of periodontal condition [62, 63]. Thus, the relationship between the severity of periodontitis and the duration of hemodialysis is until now confusing. However, this divergence in the results can be related to differences in patient ethnic and genetic backgrounds and life regimes, as well as various other factors that may have an influence on the pathogenesis and severity of periodontitis like age, medical condition regular dental care, and oral hygiene habits [64].

An important observation in our study was an absence of clinical signs of gingival inflammation with pale gingiva despite the presence of plaque deposits however with the presence of BOP. Our finding was in accordance with a previous study that found the uremic in the hemodialysis patients may suppress inflammatory reactions in the tissues leading to hiding signs of gingival inflammation [65].

The medical records of all patient’s samples reported having iron deficiency anemia with low serum ferritin levels. Correspondingly, it was reported by another study that as the chronic renal disease progresses, erythropoietin synthesis is declining also, hypothetically leading to anemia [66]. Anemia increases the bleeding tendency in uremia due to defects in platelets function, including decreased platelet aggregation along with compromised platelet adhesiveness.

There was a weak positive non-significant correlation between BOP and the duration of hemodialysis. It was suggested that the medication of the HD patients, such as anticoagulant therapy could produce increased bleeding on probing and thus might not directly reflect the level of inflammation of this group of patients [67].

There was a non-significant direct correlation between plaque score and duration of hemodialysis. This went in line with Al Wahadni and Al Omari [25] who concluded that individuals on hemodialysis might neglect oral hygiene or dental care as a whole due to spending a long time in the dialysis center. The patients on hemodialysis might have depression owing to their severe systemic illness as well as poor social level in most of the cases and thus, would show poor compliance during dental treatments and ignore oral health care. Consequently, it can be concluded that a high plaque score in this study mostly was the result of poor oral hygiene rather than the effect of uremia happened in hemodialysis patients.

Our finding of week association between periodontal indices and duration of hemodialysis was in agreement with another study that did not find any connection between dialysis duration and periodontal parameters [68]. This can be due to the short duration of hemodialysis that was maximum 2 years in this study.

Serum creatinine is a commonly used marker for the estimation of Glomerular Filtration Rate (GFR) and renal function [69]. It is undoubtedly the easiest diagnostic indicator for renal function and is readily available in laboratories [70]. Patients with chronic renal disease have elevated serum creatinine levels due to decreased renal clearance [71].

In our study, there was a significant strong positive correlation between PPD and deepest CAL and serum creatinine level. The same result was reported by another study that showed a significant association between serum levels of creatinine in patients undergoing hemodialysis and CAL [69]. In a study done by Kshirsagar and co-workers [72], patients with periodontitis had lower GFR and higher serum levels of creatinine compared to healthy individuals. Patients with untreated periodontal diseases have an increased risk to have lower GFR and subsequently elevated serum creatinine concentrations. Moreover, Naghsh et al. [69] expected that it’s possible that periodontal disease worsens renal insufficiency and decreases glomerular filtration and this leads to an increase in serum creatinine level.

There was a significant direct association between serum urea BOP, PPD, and deepest CAL. As CKD progress, there was a drop in GFR while a rise in serum levels of urea causing uremia [44]. Uremia induces immunodeficiency due to the increase of toxic waste products in the bloodstream, resulting in suppression of cellular and humoral immunity in these patients. This allows the unhindered growth of periodontal pathogens facilitating apical migration of epithelial attachment along with changes in bone mineral density and resorption of alveolar bone accelerating periodontal disease progression.

## Conclusions

In the present study, a high frequency of periodontitis was found among ESRD patients on hemodialysis being more prevalent in the severe form (stage III) periodontitis. Diabetes and hypertension were the most prevalent medical conditions.

There was an important direct correlation between the severity of periodontitis and CAL with a duration of hemodialysis. There was a weak insignificant association between periodontal indices (PD, BOP, and plaque score) and duration of hemodialysis.

### Limitations and recommendations

The cross-sectional design makes it not possible to determine the direction of the observed relationships, because the periodontal condition was only assessed once, not over time and due to the lack of the control group. As the intraosseous defect and furcation defect were not considered, all clinical tests were performed during hemodialysis sessions, which could raise the risk of bias.

These data, on the other hand, are typically ignored in epidemiological investigations. Some evaluations were hampered by the lack of ideal conditions. Because of the high prevalence of periodontitis and the sample's poor socioeconomic status, a control group without periodontitis could not be employed in the study. Furthermore, more studies need to be conducted studying the periodontal disease severity in hemodialysis patients in correlation with different durations of hemodialysis and taking into account their medical treatment that might affect the severity of periodontal conditions or may overwhelm present periodontal inflammation.

Other health professionals suggested to take part in the oral health awareness campaign. Examination of the oral cavity by nephrologists and other health care specialists, particularly in areas where patients' access to basic dental care, particularly those who rely on the national health system, is highly limited. The early detection of periodontal disease, would be an appropriate strategy in Egypt and other countries around the world, that could aid in early intervention, facilitating an overall better quality of life for ESRD on hemodialysis patients.

## Data Availability

The data that support the findings of this study are available from governmental hospitals and hemodialysis centers but restrictions apply to the availability of these data, which were used under license for the current study, and so are not publicly available. Data however available from the corresponding author upon reasonable request.

## Footnotes

1 R Core Team (2021). R: A language and environment for statistical computing. R Foundation for Statistical Computing, Vienna, Austria. https://www.R-project.org/.
